# Biofilms as “Connectors” for Oral and Systems Medicine: A New Opportunity for Biomarkers, Molecular Targets, and Bacterial Eradication

**DOI:** 10.1089/omi.2015.0146

**Published:** 2016-01-01

**Authors:** Herman O. Sintim, Ulvi Kahraman Gürsoy

**Affiliations:** ^1^ Department of Chemistry and Biochemistry, University of Maryland, College Park, Maryland.; ^2^ Department of Chemistry, Purdue University, West Lafayette, Indiana.; ^3^ Department of Periodontology, Institute of Dentistry, University of Turku, Turku, Finland.

## Abstract

Oral health and systems medicine are intimately related but have remained, sadly, as isolated knowledge communities for decades. Are there veritable connector knowledge domains that can usefully link them together on the critical path to biomarker research and “one health”? In this context, it is noteworthy that bacteria form surface-attached communities on most biological surfaces, including the oral cavity. Biofilm-forming bacteria contribute to periodontal diseases and recent evidences point to roles of these bacteria in systemic diseases as well, with cardiovascular diseases, obesity, and cancer as notable examples. Interestingly, the combined mass of microorganisms such as bacteria are so large that when we combine all plants and animals on earth, the total biomass of bacteria is still bigger. They literally do colonize everywhere, not only soil and water but our skin, digestive tract, and even oral cavity are colonized by bacteria. Hence efforts to delineate biofilm formation mechanisms of oral bacteria and microorganisms and the development of small molecules to inhibit biofilm formation in the oral cavity is very timely for both diagnostics and therapeutics. Research on biofilms can benefit both oral and systems medicine. Here, we examine, review, and synthesize new knowledge on the current understanding of oral biofilm formation, the small molecule targets that can inhibit biofilm formation in the mouth. We suggest new directions for both oral and systems medicine, using various omics technologies such as SILAC and RNAseq, that could yield deeper insights, biomarkers, and molecular targets to design small molecules that selectively aim at eradication of pathogenic oral bacteria. Ultimately, devising new ways to control and eradicate bacteria in biofilms will open up novel diagnostic and therapeutic avenues for oral and systemic diseases alike.

## Bacteria and Biofilms

Microorganisms are all around us. Their numbers are so large that when we combine all plants and animals on earth, the total biomass of bacteria is still bigger. They literally do colonize everywhere, not only soil and water but our skin, digestive tract, and even oral cavity are colonized by bacteria. Microbial communities can also impact responses to drugs and other xenobiotics (El Rakaiby et al., 2014).

As a matter of fact, the first observed bacteria by a microscope were from the oral cavity. It was a dental plaque that the father of microbiology, Anton van Leeuwenock, used as a specimen to observe bacteria under his first ever microscope. At first, when humans serendipitously learned about microorganisms, these small creatures were linked only with illness and disease. Today we know that bacteria contribute to homeostasis and regulation of human body, for example, by digesting indigestible molecules (such as nitrate) for us and converting them to useable products (such as nitrite) for us (Lundberg et al., 2008).

As stated, bacteria are everywhere, but are rarely found to be alone or floating freely (in planktonic forms). In water pipes, ships' hulls, soil, or even in the mouth of humans, bacteria prefer to bind on hard surfaces and form biofilms. Still, bacteria build biofilms only under distinctive situations, continuous sedimentation on a surface do not necessarily lead to biofilm formation (Seneviratne et al., 2012). Biofilms are sophisticated structures and to form biofilms bacteria adhere, multiply, secrete an extracellular polymeric matrix, and organize a three-dimensional community.

A typical biofilm consists of mono- or polymicrobial cells, polysaccharide, proteins, nucleic acids, and lipids (Flemming and Wingender, 2010). Biofilms are associated with nearly two third of all microbial infections in US (Potera, 1999), and should not be simply viewed or under estimated as “bacterial accumulations on surfaces”.

## Oral Biofilms

Oral biofilms carry similarities with environmental biofilms, but at the same time differ significantly. In the oral cavity, biofilms are composed of multispecies of bacteria, comprising of up to 700 different microbial species, and 100 to 200 of these species can be found in any healthy oral cavity (Kolenbrander et al., 2010). The composition of oral biofilms predominates with bacteria and extracellular matrix. Extracellular matrix is composed of deoxyribonucleic acid, proteins, polysaccharides, and lipids. Additionally, oral biofilms contain salivary glycoproteins, gingival crevicular fluid albumin, and host cell components.

Oral cavity is a nutrient rich, moist, warm environment, which is excellent for a bacterium to survive. However, there are also challenges; bacteria need to evade the flow of saliva, host antimicrobial proteins (defensins, cathelicidin, lactoferrin), variations in nutrient availabilities, pH changes, and antimicrobials, antiseptics, and antibiotics (Abiko and Saitoh, 2007). By being a part of a biofilm, bacteria become resistant to environmental stress and find necessary nutrients for growth and replicate much easily (Jakubovics and Kolenbrander, 2010).

Formation, development, and maturation of oral biofilms require bacterial colonization, interactions between bacterial cell surface adhesins and host receptors, chemical communication between bacteria, and production of extracellular matrix. Successful accumulation and multiplication of pathogenic bacteria in oral biofilms can lead to two common diseases, dental caries and periodontitis (Beikler and Flemmig, 2011). Furthermore, it is now understood that oral bacteria contribute to the initiation and/or progression of different types of cancers (Whitmore and Lamont, 2014). Hence, a clear understanding of interactions between different species of bacteria and between bacteria and the host could lead to new strategies for eliminating pathogenic biofilms and treating oral diseases.

## Supragingival and Subgingival Biofilms: Does the Localization Matter?

Bacteria adhere to both soft and hard tissues in the oral cavity. Soft tissues, epithelial cells covering the oral mucosa, have rapid turnover rates. Thus, adhered bacteria cannot stay on this shedding surface long, and are removed together with dead epithelial cells. Teeth, on the other hand, have a non-shedding surface, and therefore, serve as an excellent binding surface for the bacteria. Supragingival biofilms are found above the gingival margin, typically characterized with Gram-positive aerobe communities. Subgingival biofilms, on the other hand, are primarily formed of Gram-negative anaerobes, and are localized under the gingival margin, between the gingiva and tooth surface.

The primary nutrient for supragingival biofilm bacteria is saliva. Bacteria in supragingival biofilms can degrade salivary components easily and efficiently. Gingival crevicular fluid is the primary nutrient source for bacteria in the subgingival biofilms (Jakubovics and Kolenbrander, 2010). It is generally true that supragingival biofilms form first and subgingival biofilms afterwards. However, when formed, subgingival biofilms demonstrate independent characteristics. The environment of subgingival biofilms is anaerobic, favoring the growth of mainly Gram-negative obligate anaerobes and restrict the growth of Gram-positive facultative aerobes. Moreover, removal of supragingival biofilm demonstrates only a minor effect on the composition of subgingival biofilms (Aruni et al., 2015).

## Oral Biofilms: A Symphony of Initial, Early, and Late Colonizers

In the oral cavity, a well-cleaned tooth surface gets covered with a protein layer in just 2 hours. This layer is called acquired enamel pellicle and it contains up to 130 different salivary, serum, and cellular proteins (Dawes et al., 2015). Adhesion of bacteria to salivary pellicle is a milestone in formation of biofilms, because with this ability, bacteria form a resistance against shearing forces in the oral cavity (Rosan and Lamont, 2000).

Gram-positive aerobic bacteria, such as *Actinomyces* spp. and oral streptococci (*Streptococcus intermedius*, *S. oralis*) are the initial colonizers of the teeth surfaces. These bacteria interact with the pellicle-coated tooth surface and other bacteria as well. New bacterial adherence to immobilized bacteria is called co-adhesion, which is a form of co-aggregation. Both co-aggregation and co-adhesion happens only between compatible organisms and requires cell surface adhesins and cognate receptors. (Kolenbrander et al., 2006). Streptococcal AGI/II proteins are good example of cell-surface molecules, which mediate co-aggregation between *S. gordonii* and *A. oris* (Egland et al., 2001). *Actinomyces naeslundii* adhere to proline-rich salivary proteins with its fimbriae, which also regulates its interbacterial binding (Yeung et al., 1999).

**FIG. 1.**
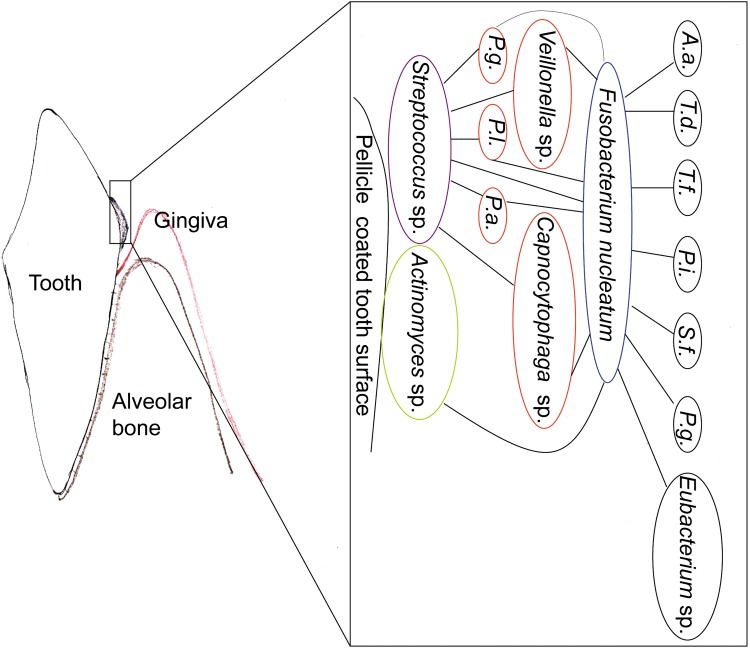
Schematic description of oral biofilms, as described by Kolenbrander et al. (2010). (*A.a.: Aggregatibacter actinomycetemcomitans, P.a.: Propionibacterium acnes, P.i.: Prevotella intermedia, P.g.: Porphyromonas gingivalis, P.l.: Prevotella loescheii, S.f.: Selenomonas flueggei, T.d.: Treponema denticola, T.f.: Tannerella forsythia*).

One major bacterial species to co-aggregate with early and late colonizers of oral biofilms is *Fusobacterium nucleatum* (Kolenbrander et al., 2010). *F. nucleatum* is a Gram-negative, anaerobic bacterium of the oral cavity, which acts as a bridge between early and late colonizers of the oral biofilms (Kolenbrander et al., 2010). Even though it is an anaerobic bacterium, it has tolerance to oxygen in biofilms (Gursoy et al., 2010). This ability allows *F. nucleatum* to support the growth of other strict anaerobes, *Porphyromonas gingivalis,* for example, in aerated environments (Diaz et al., 2002). Late colonizers of the oral biofilm are *P. gingivalis*, *Aggregatibacter actinomycetemcomitans*, *Prevotella intermedia*, *Eubacterium* spp., *Tannerella forsythia*, *Selenomonas flueggei*, and *Treponema denticola* (Fig. 1).

## Interbacterial Interactions in Oral Biofilm

Bacterial behaviors in oral biofilms are complicated. In the biofilms, bacteria compete with each other, they help each other, but very importantly they communicate with each other. Communication of bacteria was for the first time described on marine bacteria, *Vibrio fischeri*. Oral bacteria use two different systems to communicate, competence signaling peptides can only be found in Gram-positive bacteria (Suntharalingam and Cvitkovitch, 2005), while autoinducer-2 (AI-2) can be found in both Gram-positives and Gram-negatives (Sun et al., 2004).

Studies in the culture media of *F. nucleatum*, *P. gingivalis*, and *P. intermedia* reported that several strains of these bacteria produce AI-2 (Frias et al., 2001). Among the early colonizers, *S. oralis* 34 and *A. naeslundii* T14V also produce AI-2 (Rickard et al., 2008). Moreover, biofilm formation of *A. actinomycetemcomitans* is dependent of AI-2 signalling (Shao et al. 2007). Recent evidence suggested that bacterial molecules of communication not only regulates interbacterial communication, but also coordinate interactions of *P. gingivalis* with the host (Scheres et al., 2015).

Biofilms are sophisticated environments and carry evidence of mutualism and competition. A well-known example for the mutualism is the metabolic interaction between bacteria, however, exchange of signaling molecules and horizontal gene transfer also take part in it. A good example to mutualism is the interaction between oral streptococci, lactobacilli, *A. actinomycetemcomitans*, and veillonellae. Lactate, metabolic end-product of streptococci and lactobacilli, can be utilized by *A. actinomycetemcomitans*. Presence of lactate in the growth media makes growth of *A. actinomycetemcomitans* faster and stronger (Brown and Whiteley, 2007; Jakubovics and Kolenbrander, 2010). Co-aggregations and cell-to-cell contacts of *P. intermedia* and *P. nigrescens* with *F. nucleatum* induce biofilm formation of these bacteria, however this finding is not dependent on AI-2 (Okuda et al., 2012).

*P. gingivalis* is an acid-sensitive bacterium and cannot grow in conditions with low pH. *P. intermedia* and *F. nucleatum* produce ammonia and organic acid as metabolic outcomes, which lead to an increase in pH and provide a suitable growth condition to the acid-sensitive bacterium *P. gingivalis* (Takahashi et al., 2003). Cell-to-cell contact of bacteria is critical, autoaggregation of *F. nucleatum* in high cell numbers led to changes in expressions of at least 100 genes (Merritt et al., 2009).

Nevertheless, bacteria in biofilms do not only support each other, but also compete with each other. *S. mutans* and *S. salivarius* in the oral cavity produce antimicrobial peptides, bacteriocins, which can kill other bacteria (Hale et al., 2005). Additionally, oral streptococci produce hydrogen peroxide, which cause oxidative stress on strict anaerobes (Holmberg and Hallender, 1973).

## Disruption of Oral Bacteria Biofilms

**FIG. 2.**
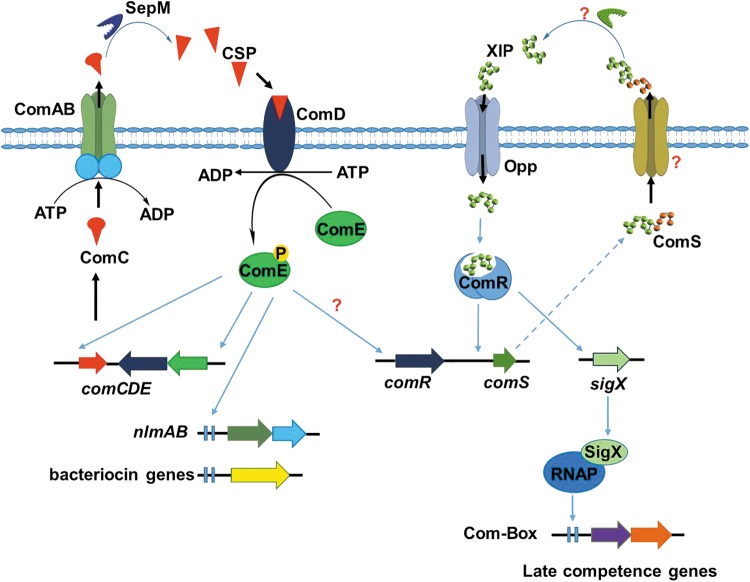
ComCDE and ComRS quorum-sensing regulatory systems in *S. mutans*. Both systems utilize peptides as autoinducers and modulate the production of bacteriocins and bacteriocin self-immunity proteins (ComCDE), biofilm formation, and competence.

**FIG. 3.**
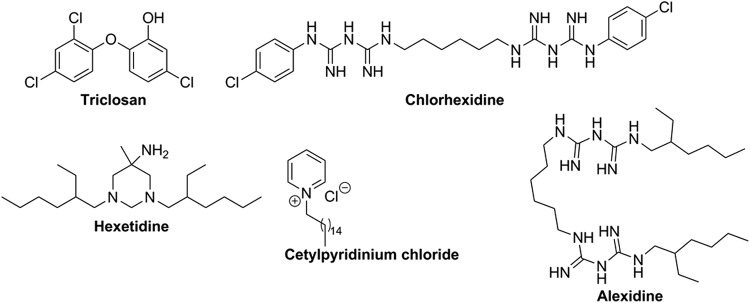
Common molecules found in mouthwashes and toothpastes with antimicrobial and anti-biofilm properties.

Three kinds of small molecules that inhibit biofilm formation by oral pathogens have been described. These are: i) inhibitors of cell-to-cell communication (quorum sensing, Fig. 2); ii) synthetic antibacterial agents that have both bactericidal and antibiofilm properties (Fig. 3); and iii) natural products, mainly isolated from food, leaves, and essential oils (Fig. 3), that either inhibit biofilm formation or disperse established biofilms.

## Inhibitors of Quorum Sensing

AI-2 and CSP are the two QS autoinducers that have been thoroughly investigated as signaling molecules amongst oral bacteria, especially in *S. mutans*. The ComCDE (Li et al., 2001) and ComRS (Mashburn-Warren et al., 2010) systems, which utilize signaling peptides ComC signal peptide (CSP) and XIP, respectively, are the two major quorum-sensing systems in *S. mutans*. CSP-mediated signaling regulates DNA release, competence, bacteriocin production, stress response, and biofilm formation.

The biofilm mass produced by *S. mutans*, which did not produce CSP (comC mutant), was lower than that produced by wild-type *S. mutans* (Li et al., 2002), highlighting the importance of CSP signaling in biofilm formation by *S. mutans*. Interestingly, higher concentrations of CSP (i.e., concentrations that are higher than what is required for quorum sensing) have been shown to kill *S. mutans* (Qi et al., 2005). Many workers have investigated the antimicrobial and antibiofilm activities of various CSP analogs, and a few were found to either have antimicrobial or antibiofilm properties (LoVetri and Madhyastha, 2010; Petersen et al., 2006). For example, CSP analog, KBI-3221 (SGSLSTFFRLFNASFTQALGK) caused reduction in *S. mutans* biofilm mass (LoVetri and Madhyastha, 2010).

AI-2 is a so-called ubiquitous quorum sensing signal that is found in both Gram-negative and Gram-positive bacteria. AI-2 has been implicated in mutualistic biofilm formation between *S. gordonii* and *S. oralis* (Saenz et al., 2012) and also between *A. naeslundii* and *S. oralis* (Rickard et al., 2006). AI-2 has also been implicated in biofilm formation by *A. actinomycetemcomitans* (Shao et al., 2007) or *S. intermedius* (Ahmed et al., 2008). Only a handful of small molecules that inhibit oral bacteria biofilm formation via the disruption of AI-2 signaling have been reported. AI-2 contributes to the co-aggregation of *F. nucleatum* with many bacteria. Interestingly, (5Z)-4-bromo-5-(bromomethylene)-2(5H)-furanone and D-ribose inhibited AI-2-induced biofilm growth and co-aggregation between *F. nucleatum* and various bacteria (*P. gingivalis, T. denticola,* and *T. forsythia*) (Jang et al., 2013).

There are few examples of oral bacteria, such as *P. gingivalis*, which respond to N-acyl homoserine lactones. Asahi et al. (2010) showed that analogs of AHL could reduce the thickness of biofilm formed by *P. gingivalis.* In another study, the same group demonstrated that AHL analogs synergistically enhanced the potencies of ofloxacin, cefuroxime, and minocycline against biofilms formed by *P. gingivalis* (Asahi et al., 2012).

## Antibacterial Agents That Have Both Bactericidal and Antibiofilm Properties Against Oral Pathogens

Antimicrobial agents or antiseptics that are found in toothpastes and mouthwashes (see Fig. 3) include fluoride, triclosan, alexidine, chlorhexidine, hexetidine, benzalkonium chloride, and cetylpyridinium chloride. Fluoride's anticaries action is mainly derived from its role in remineralization, but some evidence also point to a direct antimicrobial effect of fluoride via the inhibition of bacterial metabolism (Marquis, 1995).

Triclosan is a fatty acid synthase inhibitor and found in several products, including soaps, body washes, toothpastes, and mouth rinses. Triclosan has antiplaque activity, and has been demonstrated to kill bacteria in oral biofilms (Marsh and Bradshaw, 1993). For example, Guggenheim et al. (2001) demonstrated that triclosan is active against supragingival plaque containing *A. naeslundii*, *V. dispar*, *F. nucleatum*, *S. sobrinus,* and *S. oralis.* Despite its wide use in hygiene products, there is accumulating evidence that triclosan alters hormone regulation in animals (Paul et al., 2013). In humans, elevated exposure to triclosan is associated with increased risk of obesity (Lankester et al., 2013).

Alexidine and chlorhexidine have antimicrobial activities against several oral bacteria. Both antimicrobial agents are active against *S. mutans* biofilm (Ruiz-Linares et al., 2014). Chlorhexidine affects metabolism in bacteria by promoting the leakage of metabolites from bacterial cells (Cheung et al., 2012; Iwami et al., 1995). Chlorhexidine has been used as an adjunct to primary periodontal and endodontic treatments for decades. Long-term use of chlorhexidine, however, is restricted due to discoloration of teeth and toxic effects on oral mucosa. Moreover, recent evidence on animal studies demonstrated that long-term disinfection of oral cavity with chlorhexidine impairs the nitrate-nitrite-nitric oxide balance, and eventually leads to high blood pressure (Hyde et al., 2014).

Hexetidine is a cationic antimicrobial agent that is active against both Gram-positive and Gram-negative bacteria. It is used in medicated mouth rinses, and has both antiplaque and antigingivitis properties (Sharma et al., 2003). Cationic ammonium compounds, benzalkonium chloride and cetylpyridinium chloride (Asadoorian and Williams, 2008), have antiseptic properties and have been shown to reduce oral biofilm, as well as reducing gingivitis. These ammonium compounds probably inhibit bacterial growth via the disruption of the bacterial membrane (McDonnell and Russell, 1999). The aforementioned compounds that are found in mouthwashes and toothpastes potently kill oral bacteria or reduce plaque formation, but concerns about safety (Jones, 1997), which is an ongoing debate, have spurred the search for other compounds from natural sources that may have a more desirable toxicity profile.

## Other Small Molecules, Mainly from Natural Sources, that Either Inhibit Biofilm Formation or Disperse Established Biofilms

*S. mutans* is one of the major cariogenic bacteria, and the molecular details underlying how this bacteria attach to surfaces is now well understood. Glucosyltransferases (GTFB, GTFC, and GTFD) secreted by *S. mutans* catalyzes the formation of glucans, which attach to surfaces, via the polymerization of glycosyl units from carbohydrates (Tsumori and Kuramitsu, 1997). The bacteria then uses surface proteins, GbpA (Russell, 1979), GbpB (Smith et al., 1994), GbpC (Sato et al., 1997), and GbpD (Russell, 1979), which bind to the glucans, to adhere to surfaces. This is termed the sucrose-dependent pathway and is responsible for plaque formation.

**FIG. 4.**
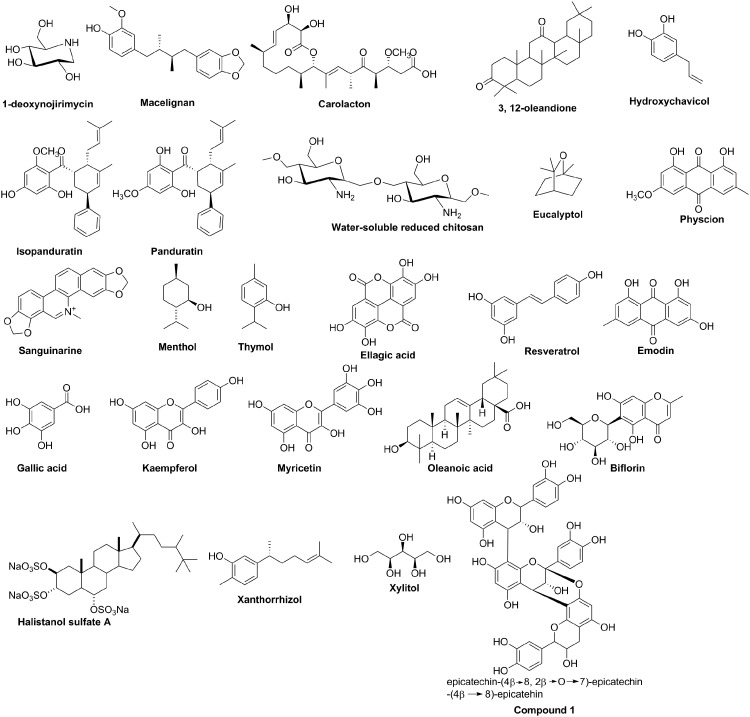
Natural products with anti-biofilm properties.

Natural products, such as polyphenols that are found in trees, are known inhibitors of *S. mutans* glucosyltransferases (Nakahara et al., 1993) and have shown antibiofilm activities against oral biofilms, *vide infra*. Several other phenols (Fig. 4) also inhibit the biofilms of *S. mutans* and other oral bacteria but via poorly defined mechansims.

Macelignan, at 10 μg/mL, reduced single primary biofilms of *S. mutans*, *S. sanguis,* and *A. viscosus* by 50% when applied for 30 min (Yanti et al., 2008). Panduratin and isopanduratin have potent activities against several oral bacteria, and inhibited formation of multispecies oral biofilm consisting of *S. mutans*, *S. sanguis,* and *A. viscosus* (Yanti et al., 2009). Hydroxychavicol is efficacious against adherent *S. mutans* in the presence of sucrose (Sharma et al., 2009). Xanthorrhizol (XTZ), which is found in the rhizome of *Curcuma xanthorrhiza* Roxb., was found to differentially affect the biofilm of *S. mutans* at different phases of growth. Whereas 5 μM of XTZ completely inhibited *S. mutans* biofilm at the adherent phase of growth, up to 50 μM of XTZ dispersed 76% of *S. mutans* biofilm at the plateau accumulated phase (Rukayadi and Hwang, 2006).

A fraction containing resveratrol, emodin, and physcion, obtained from the plant *Polygonum cuspidatum*, reduced the production of water-insoluble polysaccharides by *S. mutans* (Pandit et al., 2012). Gallic acid and methyl gallate inhibited the *in vitro* formation of S. mutans biofilm, albeit at high concentrations (1 mg/mL for gallic acid and 4 mg/mL for methyl gallate) (Kang et al., 2008). Ellagic acid inhibited the formation of water-insoluble glucan generated by *S. mutans* (Loo et al., 2010).

Thymol, found in in oil of thyme, has antiplaque activity but it is more effective when used in combination with chlorhexidine digluconate (Filoche et al., 2005). Carvacrol is a phenol isomer of thymol, and it demonstrates strong antibacterial and antibiofilm on periodontal pathogens, and antiapoptotic effects on epithelial cells (Zeidan-Chulia et al., 2014). Flavonoids such as kaempferol, myricetin, and biflorin, and proanthocyanidins (such as compound 1, see Fig. 4), commonly found in cranberry fruit, inhibited surface-adsorbed glucosyltransferases and acid production by *S. mutans* cells (Duarte et al., 2006).

In addition to the aforementioned phenolic compounds, other nonphenolic small molecules such as 1-deoxynojirimycin (Islam et al., 2008), carolacton (Kunze et al., 2010), 3,12-oleandione (Murugan et al., 2013), water-soluble reduced chitosan (Bae et al., 2006), eucalyptol and menthol (Chung et al., 2006), sanguinarine (Hannah et al., 1989), oleanoic acid (Kozai et al., 1987), xylitol (Loesche et al., 1984), and halistanol sulfate A (Lima et al., 2014) have all been shown to possess anti-caries properties.

Many of the natural phenolic compounds, including the flavonoids, tannins, coumarins, lignans, stilbenes, and quinones have long been in use in treatment of dental and medical diseases. Nevertheless, there are some issues that limit their common use. Majority of these compounds are volatile, meaning that their residual effect is short-lived. Second, these compounds require a solvent such as alcohol (or propylene glycol in non-alcohol formulas) to homogenously dissolve in oral health care product. These solvents bring additional problems, such as being risk factor of oral cancers. Finally, in animal studies it is observed that several phenolic compounds have neurotoxic and hepatotoxic effects (Ellse and Wall, 2014).

## Role of OMICS in Development of Next-Generation Anti-Infectious Agents

Despite many years of intensive research to find small molecules that can selectively target pathogenic oral bacteria without affecting commensal bacteria, there is no drug in use today that achieves selective targeting. The different OMICS technologies, especially the newer and powerful ones that can be used on large systems, could help identify targets that are critical for pathogenicity. For example, with the use of Stable Isotope Labeling by Amino Acids in Cell Culture (SILAC)-based quantitative proteomics (SILAQ) or other relative proteomics quantitation methods such as isotope-coded affinity tags (ICAT), isobaric tags for relative and absolute quantitation (iTRAQ), or metal-coded tags (MeCAT), it could be possible to identify proteins that are overexpressed in pathogenic but not commensal oral bacteria. Some of these proteins may end up being critical for periodontal disease or caries formation.

Analogously, RNA profiling of pathogenic oral bacteria at various stages of plaque formation could yield valuable insights into which gene products should be targeted for anti-infection therapy. Finally, great advances have been made to improve the sensitivity of mass spectrometry instruments, and this has now permitted the identification of small molecules in complex biological environments, and in the presence of hundreds of other small molecules. Hence the stage is set to use these sophisticated metabolomic approaches to discover novel signaling molecules that mediate bacteria perseverance. Targeting the signaling systems that use such signaling molecules could lead to new generation anti-caries agents that do not target commensal oral pathogens.

## Conclusions and Future Outlook

In the last two decades, many molecular details that underpin the co-aggregation and/or biofilm formation of oral bacteria have been well elucidated (Rabin et al., 2015a). Many of the enzymes that promote the formation of water-insoluble glucan matrices, as well as enzymes that facilitate acid tolerance by cariogenic bacteria, have been well characterized. The stage is therefore set to develop small molecules to inhibit these important enzymes that are involved in caries formation.

On the other hand, although many molecules that are commonly found in plants and food, oxidizing agents (Wennström and Lindhe, 1979), synthetic compounds such as salifluor (Coburn et al., 1981; Furuichi et al., 1996), and metals (Ingram et al., 1992; Waaler and Rölla, 1980) have been shown to inhibit biofilm formation by many oral bacteria, the molecular targets of these molecules have not been characterized (Rabin et al., 2015b). Again, we believe that omics will play an important role in unraveling the targets of many antimicrobial agents that have shown activity against oral pathogens (Bowler et al., 2013; Karaosmanoglu et al., 2014).

The veritable links between oral health and systems medicine, as noted in this review article, should ultimately help to cultivate new and sustainable innovation ecosystems in the field of omics sciences (Dandara et al., 2014). We think connecting the dots between these two knowledge domains is long overdue and the present special issue shall greatly remedy these gaps in omics scholarship.
